# Investigation of the contribution of an underlying platelet defect in women with unexplained heavy menstrual bleeding

**DOI:** 10.1080/09537104.2018.1543865

**Published:** 2018-12-06

**Authors:** Gillian C. Lowe, Roksana Fickowska, Rashid Al Ghaithi, Annabel Maclachlan, Paul Harrison, Will Lester, Steve P. Watson, Bethan Myers, Justin Clark, Neil V. Morgan

**Affiliations:** 1 Comprehensive Care Haemophilia Centre, University Hospital Birmingham NHS Foundation Trust, Birmingham, UK; 2 Institute of Cardiovascular Sciences, College of Medical and Dental Sciences, University of Birmingham, Birmingham, UK; 3 Institute of Inflammation and Ageing, College of Medical and Dental Sciences, University of Birmingham, Birmingham, UK; 4 Department of Haematology, Lincoln County Hospital, Lincoln, UK; 5 Haemostasis & Thrombosis Unit, Leicester Royal Infirmary, University Hospitals Of Leicester NHS Trust, Leicester, UK; 6 Department of Gynaecology, Birmingham Women’s and Children’s NHS Foundation Trust, Birmingham, UK

**Keywords:** Aggregometry, bleeding, heavy menstrual bleeding, platelet function defects, platelet function tests, platelets

## Abstract

Heavy menstrual bleeding (HMB) is often undiagnosed in women and can cause discomfort and distress. A haemostatic cause for excessive bleeding is often not routinely investigated and can lead to hysterectomy at an early age. A prospective cohort study was carried out to determine whether certain patients with unexplained HMB have an underlying platelet function defect (PFD). The Genotyping and Phenotyping of Platelets (GAPP) study recruited 175 women with HMB and 44 unrelated volunteers from 25 Haemophilia Centres across the UK, and a tertiary gynaecology service. Bleeding history was assessed using the International Society on Thrombosis and Haemostasis Bleeding Assessment Tool (ISTH-BAT). Platelet count, platelet size, haemoglobin and mean corpuscular volume were measured in whole blood using the Sysmex XN-1000 Haematology Analyzer. Platelet function testing using lumiaggregometry and flow cytometry was performed in patients included in this study. A PFD was identified in 47% (82/175) of patients with HMB. Cutaneous bleeding was the most frequent additional bleeding symptom (89% in PFD and 83% with no PFD). Whole blood platelet count was significantly lower (*P* < 0.0001) between the PFD group and no PFD group. The prevalence of anaemia did not differ between patients and healthy volunteers. Clinical evaluation alone is insufficient to determine presence of an underlying PFD in patients with HMB. Platelet function tests may be considered and clinical guidelines may include them in their algorithms. An appropriate diagnosis and subsequent tailored management of HMB may prevent unnecessary surgery and help manage future haemostatic challenges.

## Introduction

Heavy menstrual bleeding (HMB) has been quantitatively defined as menstrual bleeding exceeding seven days or a blood loss of more than 80 millilitres per cycle. However, such a definition is not useful clinically and does not take into account the impact of HMB on the individual. A more contemporary definition is that proposed by the National Institute of Health and Care Excellence (NICE): “excessive menstrual blood loss which interferes with a woman’s physical, social, emotional and/or material quality of life” [1]. HMB is common in women of reproductive age [2] accounting for a large proportion of health care resources in primary and secondary care [3]. Internationally, the World Health Organisation estimates that 18 million women suffer from HMB [4].

There are a variety of medical, radiological and surgical treatments for HMB but accurate diagnosis of the underlying cause is needed to optimise outcomes [3]. Several structural and non-structural causes for HMB have been identified within the International Federation for Obstetrics and Gynaecology (FIGO) PALM-COEIN classification system. Systemic disorders of haemostasis are recognised as a cause of HMB [5]. From a haematological perspective, the 2018 NICE HMB guideline update [6] recommends assessment of anaemia through the taking of a full blood count (FBC) but laboratory testing for coagulopathies such as von Willebrand’s Disease (VWD) is only recommended in selected cases, namely women who have had HMB since menarche or have a personal or family history suggesting a coagulation disorder. However, in general gynaecological practice systematic screening for potential bleeding disorders through structured history taking is not routine. Thus, the prevalence of mild bleeding disorders (MBDs) contributing to HMB may be underestimated and women may receive suboptimal or unnecessary treatment impacting adversely upon their quality of life.

Research suggests that women with PFD or coagulation defects, e.g. VWD, experience severe bleeding problems including HMB [7]. An estimated 13–20% of women with unexplained HMB have reduced VWD levels [8]. However, the frequency of other potential underlying bleeding disorders and mild platelet disorders have not been confirmed [9]. Currently, there are no guidelines addressing platelet function testing in patients with HMB [7,10]. Recent studies have suggested that investigation of an underlying platelet defect in females with unexplained HMB can optimise management and reduce rates of surgical intervention such as hysterectomy in affected women [7,10]. Thus, comprehensive diagnosis of the underlying causes of HMB, including the presence of MBDs, can optimise patient outcomes by avoiding ineffective treatments, and preventing unnecessary surgery such as hysterectomy with its associated morbidity and costs. Medical treatments can then be targeted at the underlying haematological problem which may be contributing wholly or partly to the HMB. Therapies such as inhibitors of fibrinolysis (e.g. tranexamic acid), desmopressin or platelet transfusions could be considered.

The UK-GAPP study began in 2006 when the goal was to perform functional and genetic studies on patients with clinically diagnosed platelet-based bleeding disorders of unknown cause. It has recruited over 850 patients from 25 collaborating Haemophilia Centres and from a tertiary gynaecology service [11]. A proportion of the female participants have unexplained HMB and may have undiagnosed platelet function defects (PFD) which account for their symptoms. This study aimed to analyse the data of patients with HMB within the GAPP study to determine whether a significant proportion of patients with HMB have a previously undiagnosed PFD.

## Methods

### Population

Women were recruited from participating UK Haemophilia Centres and a specialist gynaecology service at the Birmingham Women’s Hospital. Women were eligible to participate in the GAPP study (2006–2017) if they were: aged 16 years or older; referred to secondary care with the possibility of an underlying bleeding disorder based on their clinical history and were willing to take part in the study and were capable of providing informed consent. Women with HMB who had no uterine structural abnormalities (e.g. fibroids or polyps on pelvic ultrasound) presenting to a specialist gynaecology service were also invited to participate. Women were excluded if they had: no history of HMB; taken drugs which could influence platelet function within seven days of enrolment; been previously diagnosed with a bleeding disorder such as VWD; undergone major surgery in the last 6 months; chronic renal failure needing dialysis; a platelet count outside the range 100 000–450 000/μL; severe anaemia (haemoglobin (Hb) <80 g/L). In order to establish standardised reference ranges for laboratory parameters of platelet function, blood was taken from a group of consenting healthy volunteers to act as controls.

The UK-GAPP study was approved by the National Research Ethics Service Committee of West Midlands – Edgbaston (06/MRE07/36) and participants gave written informed consent in accordance with the Declaration of Helsinki. The GAPP study was registered at www.isrctn.org as #ISRCTN 77951167 and is included in the National Institute of Health Research Non-Malignant Haematology study portfolio, (ID-9858).

## ISTH BAT

The International Society on Thrombosis and Haemostasis Bleeding Assessment Tool (ISTH-BAT) was used to provide an objective evaluation of the patients bleeding history. This score was developed in an attempt to standardise history taking in patients with excessive bleeding. A score of 4 or over is considered to be elevated [12]. The ISTH bleeding scores were generated by an experienced clinician before platelet function tests were performed to avoid biased interpretation of the bleeding history following lumiaggregometry [13]. Further studies were performed to assess the platelet defects and their severity. The patients with platelet function defects were grouped based on the type of platelet defect detected by lumiaggregometry and their ISTH-BAT scores were compared.

### Blood Cell and Platelet Counts and Morphology

Bleeding severity is related to platelet count and platelet function. Therefore, platelet counts and morphology were measured from patients in whole blood using the Sysmex XN-1000 Haematology Analyzer (n = 103) (Sysmex, Milton Keynes, UK) and other methods. The PLT-F channel was used to determine platelet counts in whole blood. The haemoglobin (Hb) concentration, mean corpuscular volume (MCV) and mean platelet volume (MPV) were measured and compared between the groups. MPV, a machine-calculated measurement of the average size of platelets found in blood, tends to correlate with platelet function and is a good marker of platelet activation and reactivity [14,15], was determined from the impedance PLT-I channel. All samples were processed in tandem with travel controls, which are taken from healthy volunteers and transported and tested at the same time as participants. In addition to the measurement of Hb to indicate anaemia the MCV was also tested to give the average volume of red cells. Instrument performance was ensured by the use of daily internal quality controls (XN check, Sysmex, Milton Keynes, UK) and monthly external quality controls (UKNEQAS, Watford, UK).

### Platelet Preparation and Platelet Function Testing

Previous studies by the UK-GAPP study group have demonstrated the applicability of using light transmission aggregometry (LTA), including lumiaggregometry for testing patients with a suspected PFD to account for their bleeding symptoms [11,13,16,17]. Lumiaggregometry was used to simultaneously test platelet aggregation and secretion, for investigation of Platelet Rich Plasma (PRP) samples having platelet counts exceeding 1 × 10^8^/mL and an in-house flow-cytometry assay to assess platelet function in patients having platelet counts in PRP of less than 1 × 10^8^/mL as LTA is not reliable for low platelet counts [16]. The patients tested were found have a platelet defect using a rationalised platelet agonist panel (as outlined in Dawood et al. 2012 and Tables I and 2) choosing dose points from previous work on healthy volunteers in the GAPP study [13,16,17]. To consider the frequency of any abnormal results/platelet responses in the healthy control population we performed platelet aggregation and secretion in 68 healthy volunteers (Supplementary figure 1). The higher concentrations of agonists resulted in narrow reference intervals, but lower agonist concentrations (including some routinely used in current laboratory practice, such as ADP 3 µM and collagen 1 µg/mL) were significantly more variable.

**Table I.** **The streamlined panel of agonists used for lumi-aggregometry in diagnosing platelet function defects in 175 women with HMB**. ATP secretion from dense granules was measured with ADP 30 µM, adrenaline 10 µM, arachidonic acid 1 mM, PAR1 – specific peptide 100 µM and collagen 3 µg/ml.

Agonist	Concentration	Comments/further guidance
ADP	10 µM	Maximal, sustained aggregation expected at this concentration. If reversible aggregation seen use 30 µM.
Adrenaline	10 µM	Biphasic aggregation expected. If aggregation absent or reduced use 30 µM.
Arachidonic acid	1 mM	Maximal, sustained aggregation expected. If aggregation reduced or reversible use U46619 3 µM.
PAR-1 receptor specific peptide (SFLLRN)	100 µM	Maximal, sustained aggregation expected. If aggregation reduced use PAR-4 receptor specific peptide (AYPGKF; 500 µM).
Collagen	1 µg/ml	If aggregation reduced use 3 µg/ml. If this concentration shows reduced aggregation, use collagen related peptide (CRP) 3 µg/ml.
Ristocetin	1.5 mg/ml	Maximal, sustained agglutination (often biphasic) expected. If reduced consider vWF profile and GPIb levels by flow cytometry.

**Table II.** Typical lumi-aggregometry findings in the commonly encountered platelet defects within the HMB cohort.

Platelet defect	Aggregation results	Secretion results
Secretion defects	Absence of secondary wave aggregation to most agonists used in low concentrations, and epinephrine at all concentrations	Significantly reduced levels of ATP secretion (when normalized for platelet count)
Thromboxane pathway defects	Absence/severe reduction of aggregation in response to arachidonic acid while response to thromboxane mimetics is preserved (for cyclooxygenase defects) or absence of aggregation response to both arachidonic acid and thromboxane mimetics (for thromboxane receptor defects) Commonly, absence of secondary wave aggregation to most agonists used in low concentrations, and epinephrine at all concentrations	Normal
Gi-coupled receptor defects	Reduced aggregation to ADP with notable deaggregation even at high concentrations of agonist Reduced primary wave response to epinephrine and absence of secondary wave	Normal

In diagnosing whether the patient had a platelet function disorder, we made a judgment on their clinical bleeding history in combination with their platelet responses patterns. These are based on the higher agonist doses used in terms of their maximal amplitude and temporal response patterns for each agonist. A demonstrable platelet defect was observed in patients with abnormal aggregation and/or secretion responses and defects mostly fell into the categories of dense granule secretion defect, Gi pathway signalling defect and cyclooxygenase pathway defects. The platelet granule secretion was tested by assessment of the ATP-secretion levels in the PRP using a luciferase assay as previously described [13]. All blood tests and assays were performed within 4 hours of blood collection as previously studied [17]. Furthermore, the effect of distance travelled for transporting samples over 100 miles was not found to alter platelet aggregation responses (Supplementary Figure 2).

### Statistical Methods

Scatter dot plots were constructed to show the spread of data within each patient group with horizontal bars indicating the median and interquartile ranges. Statistical analysis between groups was performed using parametric unpaired *t*-tests for normally distributed data. Where multiple comparisons between the mean of each groups with others, a parametric ordinary one-way ANOVA with Tukey multiple comparisons test was used. For non-normally distributed data, a non-parametric Kruskal-Wallis test was used to compare means and Dunn’s adjustment applied for multiple comparisons to compare the mean rank of each group with the mean rank of every other group. An ordinary two-way ANOVA with Sidak’s multiple comparisons test was used to compare between the patient groups with and without a platelet defect.

## Results

**Figure 1.**
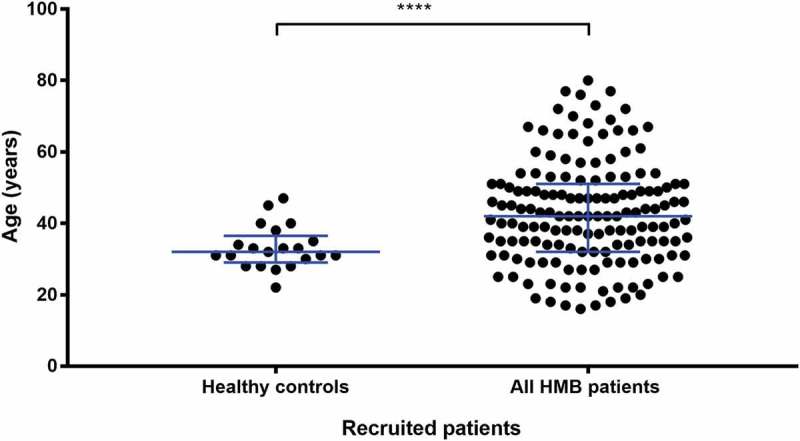
Spread of ages for all recruited patients. A scatter dot plot showing the spread of ages of all recruited patients in this study. The patients age was available for 22 healthy controls and 154 HMB patients. Horizontal bars indicate median and interquartile range. Statistical analysis was performed using parametric unpaired t-test, statistically significant difference is denoted by **** *P* < 0.0001.

A total of 175 women with a history of HMB as a primary bleeding complaint and 44 unrelated healthy volunteers were analysed in this study (Figure 1).The median age of the recruited patients with HMB was 42 years and the interquartile range (IQR) was 32 to 51 years. Over a quarter of the patients were in the age range of 41–50 (27%) followed by: over 50 years (26%), 31–40 years (25%), 20–30 years (16%) and less than 20 years (5%). The healthy volunteers forming the control group were younger with a median age of 32 years and the IQR was 22 to 36.5.

### Identification of Patients with Bleeding History and Platelet Defects Using ISTH-BAT and Lumiaggregometry

All women had a bleeding history taken at the point of inclusion into the study. In addition to HMB, most patients suffered from a variety of bleeding symptoms including epistaxis, cutaneous bruising, bleeding from minor wounds, haematuria, gastrointestinal bleeding, oral cavity bleeding, bleeding after tooth extraction, surgery, major trauma or child birth (Table III). ISTH-BAT scores were available in all 175 women with HMB and for 22/44 (50%) of the healthy controls. Lumiaggregometry identified platelet defects in 82/175 (47%) women (Table III). Statistical analysis performed using two-way ANOVA with Sidak multiple comparisons test showed no significant difference (*p* > 0.05) in any of the bleeding symptoms between the patient groups with and without a platelet defect.

**Table III.** **Phenotypic symptoms of 175 patients recruited to the UK-GAPP study with HMB of unknown aetiology**. Types of bleeding symptoms were assessed using the ISTH-BAT and the percentage of patients suffering from a platelet defect or no platelet defect using lumiaggregometry are listed. Statistical analysis was performed using ordinary two-way ANOVA with Sidak’s multiple comparisons test. P-values are listed, no statistical significance was noted (*P* ≥ 0.05).

Type of bleeding	Patients with platelet defect (n = 82)	Patients with no platelet defect (n = 93)	Percentage of total patients with bleeding tendencies	*P*-value
Heavy menstrual bleeding	82	93	100	0.9898
Cutaneous bleeding	73	77	85.7	>0.9999
Oral cavity bleeding	66	69	77.1	>0.9999
Bleeding from minor wounds	55	57	64.0	>0.9999
Bleeding after tooth/teeth extraction	55	48	58.9	>0.9999
Epistaxis	48	45	53.1	>0.9999
Post-partum hemorrhage	38	45	47.4	0.9998
Bleeding after surgery/major Trauma	44	33	44	0.9898
Gastrointestinal bleeding	25	28	30.3	>0.9999
Hematuria	18	25	24.6	0.9998
Muscle hematomas or hemarthrosis (spontaneous)	14	10	13.7	>0.9999
Other bleeding^1^	66	38	59.4	0.2343

^1^other bleeding episodes includes: venipuncture and ovulation bleeding, conjunctival hemorrhage or excessive bleeding following or venipuncture. Overall some patients had > 1 other bleeding complaint

### Systemic Bleeding Severity, Heavy Menstrual Bleeding and Platelet Defects

**Figure 2.**
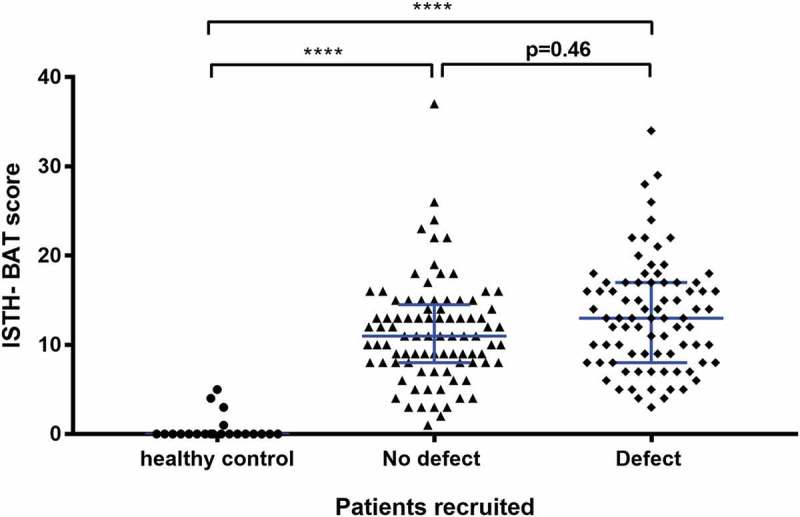
The relationship between presence of a platelet function defect detected by lumiaggregometry and the ISTH-BAT score. A scatter dot plot showing the spread of ISTH-BAT scores in the healthy controls (n = 22), no platelet defect (n = 89) and platelet defect (n = 82) groups. Horizontal bars indicate median and interquartile range. Statistical analysis was performed using the non-parametric Kruskal-Wallis test and Dunn’s adjustment for multiple comparisons, the mean rank of each column was compared with the mean rank of every other column. Statistically significant difference is denoted by **** = *P* < 0.0001. No statistical significance (*P* = 0.46) was seen between the no platelet defect and platelet defect group.

The ISTH-BAT scores were compared amongst the control, no platelet defect and platelet defect groups in order to assess whether the severity of bleeding varied between women with and without HMB and between women with HMB according to the presence of a platelet defect (Figure 2). The median ISTH-BAT score for the control group was 0 indicating no bleeding symptoms and the 95th percentile was 4.9. The median overall score for the HMB group was 12 (IQR 8–16), which was significantly higher than the control women without HMB (*P* = < 0.0001). The median ISTH-BAT scores did not differ significantly between those HMB women according to the presence of a detectable platelet defect; the HMB platelet defect group was 13 (IQR 8 to 17) compared to a median ISTH-BAT score for the HMB no defect group of 11 (IQR 8 to 15) (*P* = 0.46). Similarly, no differences between women with or without platelet defects were observed when the ISTH-BAT score calculation was restricted to the severity of bleeding in relation to menstruation (i.e. HMB alone) (median HMB score 3, IQR 2 to 4 vs. median HMB score 3, IQR of 2 to 4, respectively).

**Figure 3.**
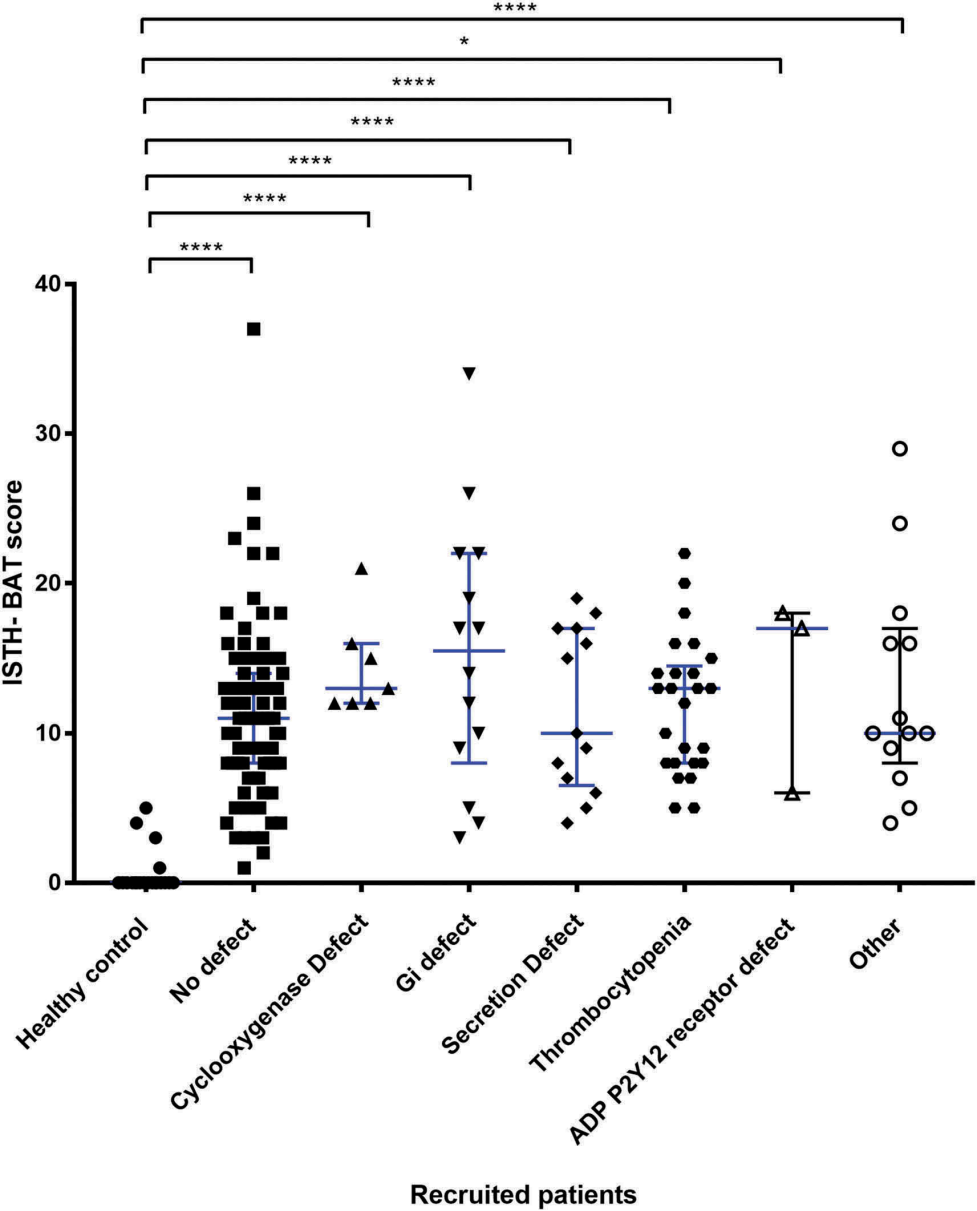
The relationship between the type of platelet defect identified by lumiaggregometry and the ISTH-BAT score. A scatter dot plot showing the spread of ISTH-BAT scores between the platelet defects identified on lumiaggregometry, healthy controls (n = 21), No defect (n = 88), defect (n = 75) of which; cyclooxygenase defect (n = 7), G_i_ receptor signalling defect (Gi defect) (n = 14), Secretion defect (n = 13), thrombocytopenia (n = 25), ADP receptor defect (n = 3) and other (n = 13). Horizontal bars indicate median and interquartile range. Statistical analysis was performed using non-parametric Kruskal-Wallis and Dunn’s multiple comparisons test, the mean rank of each column was compared with the mean rank of every other column. Statistically significant difference is denoted by * = *P* < 0.05, **** = *P* < 0.0001, no statistically significant difference (*P* > 0.05) was observed between the platelet defect subgroups.

**Figure 4.**
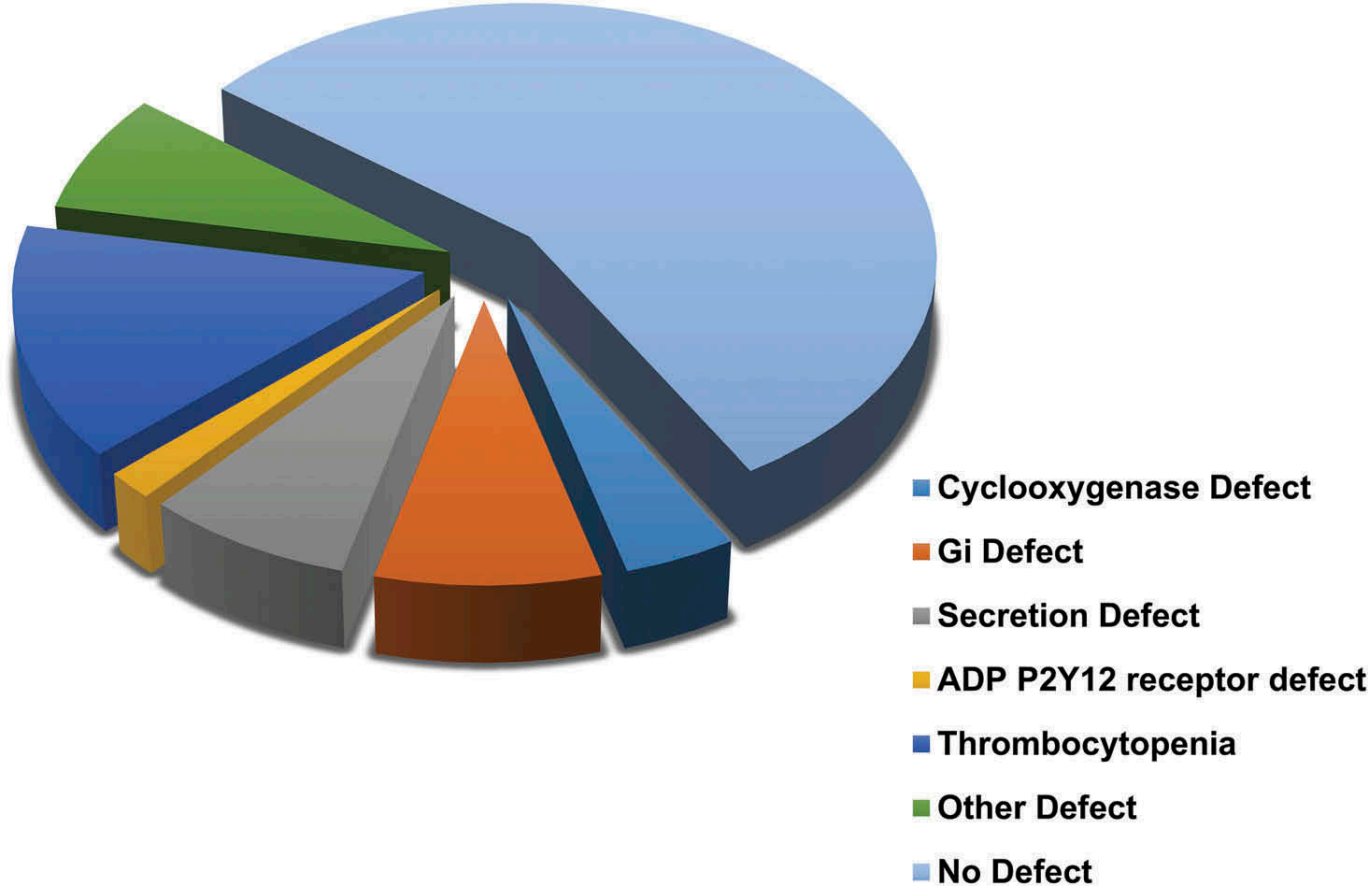
The type of platelet defect identified in the HMB group as defined by lumiaggregometry.

In the 82 women where platelet function defects were detected by lumiaggregometry, the type of platelet defect was sub-grouped into six distinct categories; cyclo-oxygenase deficiency (10%), G_i_ receptor signalling defect (20%), secretion defect (18%), thrombocytopenia (30%), ADP P2Y12 receptor defect (4%) and “other” defects (18%). The latter group of women with HMB had abnormal lumiaggregometry but without a clearly identified platelet defect. The association between the systemic bleeding severity as estimated by the ISTH-BAT scores was then compared according to the type of platelet defect (Figure 3). The groups of patients identified by lumiaggregomtery are also shown independent of the BAT score (Figure 4). The ISTH-BAT scores were not available for three patients.

The most common finding was thrombocytopenia (100–150 × 10^9^/L), which was found in 14% (n = 24/175) of HMB patients. Of the specific platelet function defects detected, women with thrombocytopenia had the lowest median ISTH-BAT score = 10 (IQR 8 to 13.5) along with platelet secretion defects (median ISTH-BAT score = 10, IQR 6.5 to 17). An ADP receptor defect was the least commonly detected defect, seen in 2% (n = 3/175) of all women with HMB but the anomaly was associated with highest median ISTH-BAT score = 17 (IQR 6 to 18). The 8% (n = 13) of women with abnormal lumiaggregometry fell into the “other” category, which contained women without a clearly identified platelet defect; the median ISTH-BAT in this group was 10 and the IQR was 8 to 17 (Figure 3).

**Figure 5.**
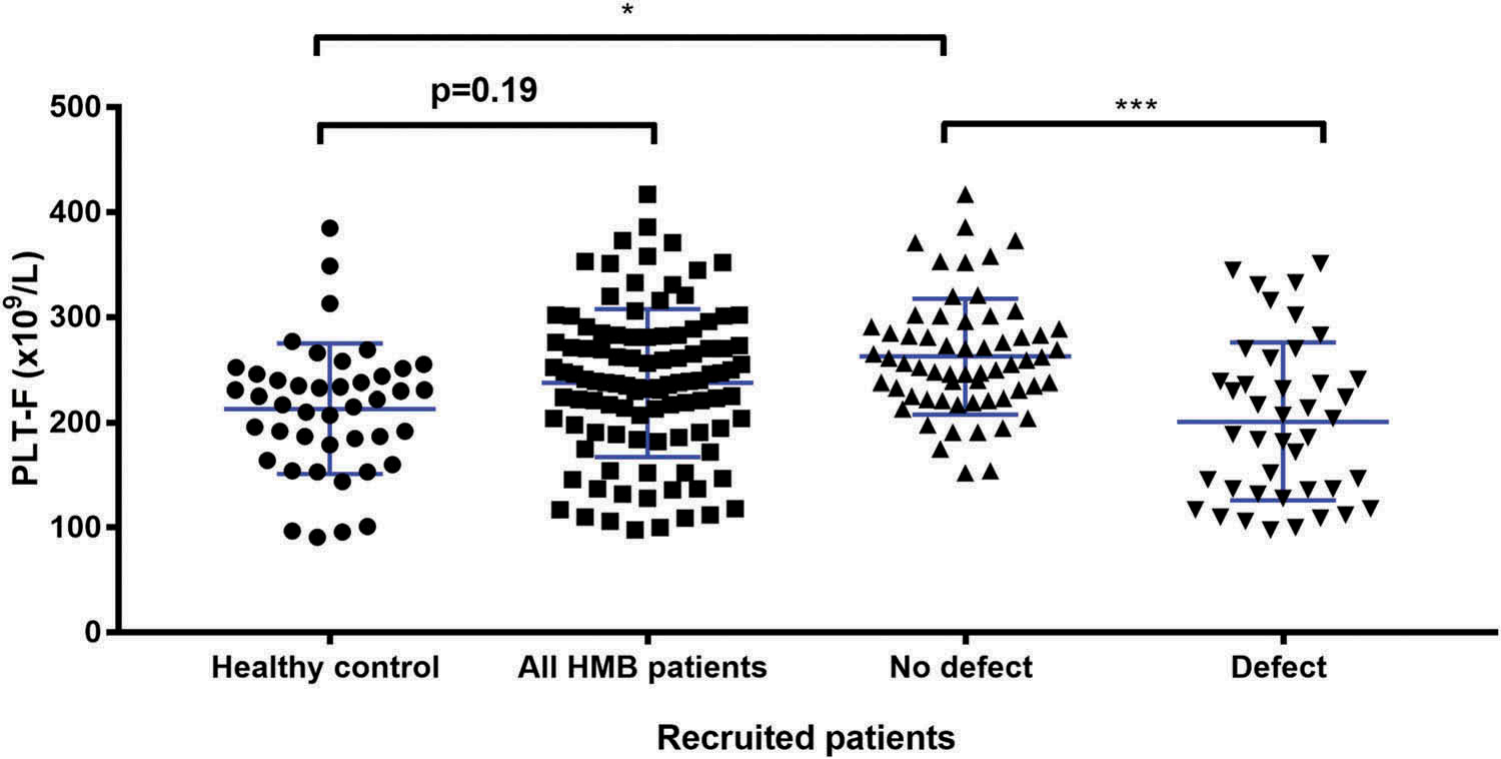
Comparison of PLT-F between types of platelet defect groups identified by lumiaggregometry. A scatter dot plot showing the spread of PLT-F results between the recruited patients. Healthy controls (n = 43), all HMB patients (n = 103), No defect (n = 61), and defect (n = 42). Error bars represent mean ± 1 SD. Statistical analysis was performed using parametric ordinary one-way ANOVA with Tukey multiple comparisons test, the mean rank of each column was compared with the mean rank of every other column. Statistically significant difference is denoted by * = P ≤ 0.05, *** = P ≤ 0.001.

### Other Laboratory Findings

Additional parameters were measured in each patient’s blood samples to further determine the possibility of an underlying platelet function defect. As bleeding severity is related to platelet count and platelet function whole blood platelet counts using PLT-F are shown for all recruited patients (Figure 5). The mean for the healthy control group of women without HMB was not significantly lower at 213.1 × 10^9^/L compared to the mean of all women reporting HMB, which was 237.5 × 10^9^/L, *P* = 0.1943). In women with HMB and no platelet defect identified by lumiaggregometry, the mean PLT-F was significantly higher at 262.8 × 10^9^/L compared to 201 × 10^9^/L in HMB women with a platelet defect (*P* < 0.001).

**Figure 6.**
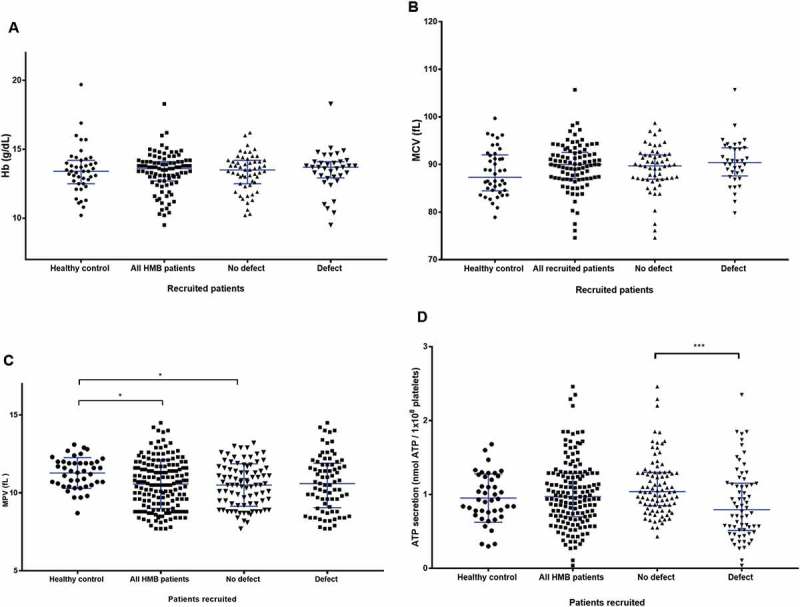
Other haematological and platelet function testing findings between the groups of patients in the study. (A) Comparison of haemoglobin between healthy volunteers and recruited patients with and without a platelet defect. A scatter dot plot showing the spread of Hb results between the recruits. Healthy controls (n = 43), all HMB patients (n = 92), no defect (n = 56), and defect (n = 36). Horizontal bars indicate median and interquartile range. Statistical analysis was performed using non-parametric Kruskal-Wallis and Dunn’s multiple comparisons test, the mean rank of each column was compared with the mean rank of every other column. Comparison between pairs of groups was not significant (*P* >0.05) for all data points. (B) Comparison of MCV parameters between the healthy control and recruited patients with and without a platelet defect. A scatter dot plot showing the spread of MCV results between the recruits. Healthy controls (n = 42), all recruited HMB patients (n = 92), No defect (n =56) and, defect (n = 36). Horizontal bars indicate median and interquartile range. Statistical analysis was performed using non-parametric Kruskal-Wallis and Dunn’s multiple comparisons test, the mean rank of each column was compared with the mean rank of every other column. Comparison between pairs of groups was not significant (*P* ≥ 0.05) for all data points. (C) Comparison of MPV parameters between types of platelet defects identifiedvia lumiaggregometry. A scatter dot plot showing the spread of MPV results between the recruits. Healthy controls (n = 43), all HMB patients (n = 162), No defect (n = 86), and defect (n = 76). Horizontal bars indicate median and interquartile range. Statistical analysis performed using the non-parametric Kruskal-Wallis test and Dunn’s adjustment for multiple comparisons, the mean rank of each column was compared with the mean rank of every othercolumn. Statistically significant difference is denoted by * = *P* ≤ 0.05. (D) Comparison of ATP-secretion between types of platelet defects identified by lumiaggregometry. Scatter dot plot showing the spread of ATP secretion results between the recruited patients. Healthy controls (n = 42), all HMB patients (n = 155), no defect (n = 89), and defect (n = 66). Horizontal bars indicate median and interquartile range. Statistical analysis was performed using non-parametric Kruskal-Wallis and Dunn’s multiple comparisons test, the mean rank of each column was compared with the mean rank of every other column. Statistically significant difference is denoted by *** = *P* ≤ 0.001.

The observed differences in PLT-F between women with and without HMB and those HMB affected women with and without platelet defects could not be explained by the presence of anaemia. The median haemoglobin (Hb) level for the control group of women without HMB was 134 g/L (IQR 125 to 142 g/L) and 136 g/L (IQR 127 to 141g/L) in women with HMB (Figure 6a). The median Hb did not differ between women with HMB according to the presence or absence of a platelet defect (135 g/L, IQR of 126 to 142 g/L vs. 137 g/L, IQR of 129 to 141 g/L. In the absence of anaemia, red blood size was tested and found to be within normal limits. Analysis of the mean cell volume (MCV) in the different groups showed no differences between women with and without HMB (87.3fL, IQR 84.5 to 92fL vs. 90.0fL, IQR 87.0 to 92.6 fL; *P* = 0.3427)) and women with HMB according to the presence or absence of a platelet function defect (90.4 fL, IQR 87.3 to 93.6 fL vs. 89.7 fL, IQR 86.9 to 92 fL respectively; *P* > 0.05) (Figure 6b).

### Mean platelet Volume

The median MPV was significantly lower in women with HMB compared to those women without HMB (healthy controls) (10.5 fL, IQR of 9.3 to 11.7 fL vs. 11.4 fL, IQR 10.5 to 12 fL respectively; *P* = 0.0199) (Figure 6c). No differences in MPV were seen in women with and without detectable platelet defects 10.5 fL, IQR of 8.9 to 11.9 fL1vs. 0.6 fL, IQR 9.3 to 11.63 fL, respectively. The median MPV of the platelet defect group was 10.5 fL with an IQR of 8.9 to 11.9 fL. A significant decrease (*P* < 0.05) in MPV levels were seen in all patients with HMB and no platelet defect in comparison to controls (Figure 6c).

### Other Platelet Investigations

Further assessments of platelet function were tested. The immature platelet fraction for all recruited women fell within the normal healthy range and no significant differences were observed between women with and without HMB or women with HMB and a platelet function defect or not. The median value of platelet ATP-secretion (a marker of platelet activation) in the healthy control group was 0.925 nmol ATP/1 × 10^8^ platelets (IQR 0.75 to 1.243 nmol ATP/1 × 10^8^ platelets), which was not significantly different from the median ATP secretion in women with HMB which was 0.97 nmol ATP/1 × 10^8^ platelets (IQR 0.7 to 1.29 nmol ATP/1 × 10^8^ platelets) (Figure 6d). In contrast, a significant difference in median ATP-secretion levels was apparent between women with HMB and a platelet defect (0.77 nmol ATP/1 × 10^8^ platelets, IQR was 0.49 to 1.14 nmol ATP/1 × 10^8^ platelets) and those women with HMB and no platelet defect (1.04 nmol ATP/1 × 10^8^ platelets, IQR 0.85 to 1.3 nmol ATP/1 × 10^8^); *P* < 0.001.

## Discussion

### Main Findings

This is one of the few studies to consider a large population of patients with unexplained HMB with a previously undiagnosed platelet function defect (PFD). We demonstrated that 47% of women reporting HMB had an underlying PFD. The ISTH-BAT found no differences in bleeding symptoms precluding the ability to clinically discriminate between women with HMB who have, or do not have, a platelet defect. Furthermore, no differences were observed in the severity of bleeding between the “no platelet defect” and “platelet defect” groups. The absence of anaemia is not surprising, however, because significant iron deficiency anaemia is uncommon in women with HMB in the developed world in the absence of uterine pathology such as fibroids [18]. It is also possible that women had received treatment for iron deficiency. The most common additional bleeding symptoms in women complaining of HMB were cutaneous bleeding and bleeding from minor wounds in keeping with the findings from previous publications [19].

### Strengths and Limitations

Several studies have studied the prevalence of von Willebrand’s disease in association with HMB [8,20] but few studies have systematically investigated PFD in women presenting with HMB and their relative clinical impact on HMB and generalised bleeding complaints. Furthermore, in contrast to previous studies [12], the ISTH-BAT score for HMB in isolation was assessed in addition to the total ISTH-BAT score, to determine the severity of HMB. Regardless of the presence of a PFD, the median ISTH-BAT score for the HMB category was 3 out of 4. A score of 3 indicates that the bleeding symptoms are severe, involving the potential need for iron therapy, time off work or school and the potential requirement to combine treatments with antifibrinolytics and hormonal therapy, aimed to reduce the menstrual flow. Phillip et al. suggested that annually 5% of women of reproductive age seek medical attention for their HMB, however, given the severity of HMB identified in this study the figure is likely to be much higher [21].

The cohort of patients assessed in this study is a hospital-based population instead of patients recruited from primary care such that most of the patients have already been treated for anaemia and their bleeding is partially managed which could affect the rate of suspected PFD. The impact of HMB and PFDs on iron deficiency may have been underestimated in our study because ferritin levels to assess iron stores were not evaluated. Furthermore, the observed frequency of abnormal platelet test results may be biased by the recruitment of subjects from haemophilia centres and may not necessarily reflect the general population of women with HMB. The ISTH-BAT scores were only available for 50% of all the healthy volunteers. The group where the BAT questionnaire was taken was done on local volunteers whereas the remaining controls were taken from remote sites where routine BAT scores are not taken.

The characteristics of platelet function disorders remain unclear and it is commonly assumed that VWD is one of the most common bleeding disorders amongst HMB patients. The patients were recruited to the GAPP study from hemophilia centres and a tertiary gynaecology service on the basis that they did not have VWD as stated in the exclusion criteria, therefore, VWD parameters were not re-tested in this study. This is a potential weakness given the high prevalence of VWD amongst HMB suffers. Furthermore the accuracy and precision of the platelet function tests are uncertain. Platelet function defects may have been undetected by lumiaggregometry if the recognised type of defect was below the sensitivity levels of the tests used. It is well known that a proportion of patients with secretion defects have normal LTA. In order to overcome the limitation of using LTA alone, LTA was combined with the assessment of ATP-secretion in the method of lumiaggregometry. In addition, PFDs may have been present that we did not test for, such as defective procoagulant activity, disorders such as these require highly specialist testing beyond the scope of this study. Alternatively, they could have a defect in another part of the coagulation pathway such as fibrinolytic defects. In light of the bleeding history of these patients, a comprehensive evaluation of potential abnormalities in the entire haemostatic pathway should be performed when technically and practically feasible.

### Interpretation

The clinical assessment, laboratory evaluation and clinical management of suspected PFD amongst HMB patients is challenging. It has been suggested that as little as 8% of women with bleeding abnormalities undergo haemostatic evaluation [21] and that 47% to 69% of patients with MBDs will remain undiagnosed [22]. Heavy menstrual bleeding is common and our study shows that nearly half of women with this complaint who do not have VWD may have a PFD. Thus, it is important to make gynaecologists aware of PFDs as a potential contributory cause of unexplained HMB. There is an increasing recognition that accurate diagnosis of the underlying cause of HMB is essential to tailor treatment and optimise clinical outcomes [5,6]. Therefore, where a woman presents with HMB and a MBD, such as Type I VWD or a suspected platelet function anomaly, referral to a haematologist or specialist joint gynaecological-haematology clinic should be considered for further management. In such patients, if VWD or an identifiable platelet defect is not present it is also important to consider further defects in other parts of the haemostatic pathway such as fibrinolysis, collagen exposure and vessel wall function, which are not routinely investigated even in more specialised laboratories. Furthermore, a recent study by Eising et al. reported a high prevalence of reduced thrombin generation and/or decreased platelet response in women with unexplained heavy menstrual bleeding, implicating the need for more specialised laboratory tests [23].

The prevalence of PFDs in our study may support the contention of other authors that platelet function testing could be incorporated into the investigative algorithms of women with unexplained HMB [8,24]. The integration of these findings into existing guidance or to inform the development of new HMB guidelines may encourage more effective non-surgical management with an emphasis on haemostatically orientated interventions such as the use of desmopressin, inhibitors of fibrinolysis (e.g. tranexamic acid) or platelet transfusions, in isolation or in conjunction with recommended, evidence-based hormonal treatments. In this way patient outcomes may be improved and the rates of more morbid surgical treatments, such as hysterectomy reduced.

### Conclusion and Research Recommendations

Previously undiagnosed platelet function disorders are highly prevalent amongst patients with HMB of an otherwise unknown cause. The comprehensive laboratory and clinical data obtained in this study have highlighted the lack of difference in clinical and routine haematological tests (full blood count) between women with a platelet function defect and women with no identifiable platelet function defect. It is evident that further platelet function tests are useful for detection of an underlying PFD. The impact of recognition of a PFD amongst women with HMB on subsequent management and clinical outcomes needs further study. It should also be borne in mind that their identification may also have a large impact on the management of their disorder in non-gynaecological situations involving future haemostatic challenges such as surgery or childbirth.
