# Supplementary material for: Investigation of the contribution of an underlying platelet defect in women with unexplained heavy menstrual bleeding
Source: Platelets. 2018 Dec 6;30(1):56–65. doi: 10.1080/09537104.2018.1543865 (PMC6406209; doi:10.1080/09537104.2018.1543865)
Supplement: Supplemental Material [file IPLT_A_1543865_SM0752.zip › supp_mat/Lowe et al Supplementary file final.pdf]

Supplementary Figures

Assessment of Platelet Aggregation and secretion in healthy volunteers.

A.

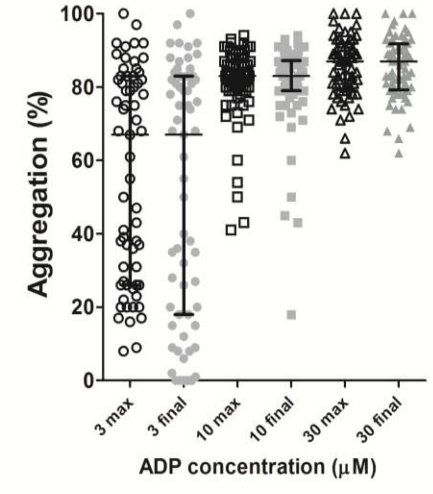

B.

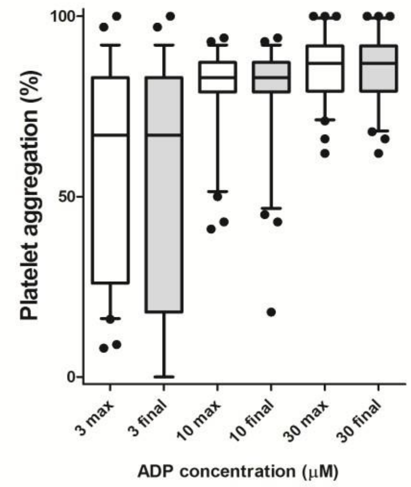

Supplementary Figure 1.1 ADP responses. For panel A, bars represent median and interquartile range. Black symbols represent maximal aggregation and grey symbols represent final aggregation 5 minutes after agonist addition. For panel B, boxes represent median and interquartile range, whiskers represent 5th and 95th percentiles and outlying points are represented by dots. Black symbols represent maximal aggregation, grey symbols represent final aggregation 5 mins after agonist addition.

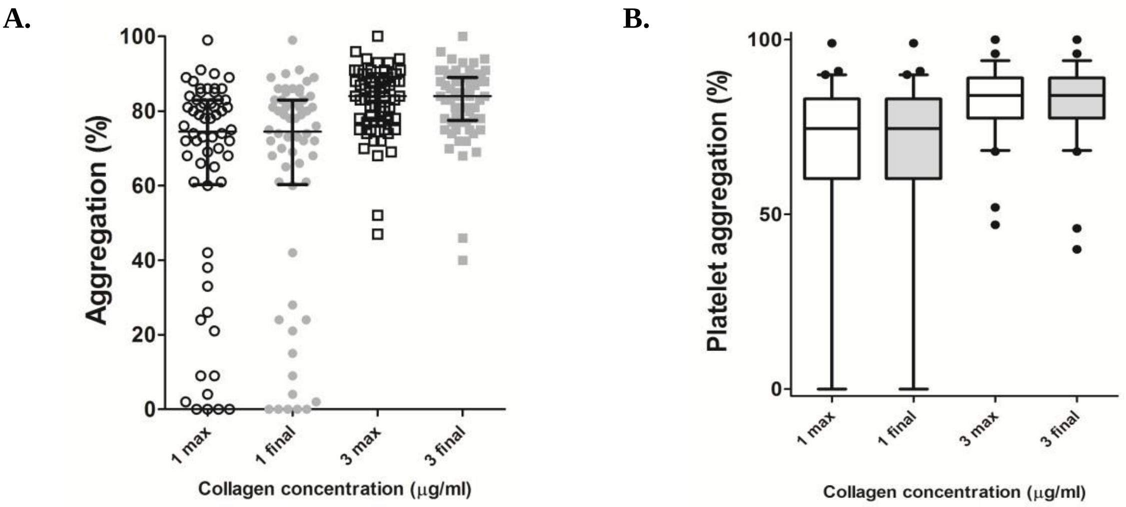

Supplementary Figure 1.2 Collagen responses. For panel A, bars represent median and interquartile range. Black symbols represent maximal aggregation and grey symbols represent final aggregation 5 minutes after agonist addition. For panel B, boxes represent median and interquartile range, whiskers represent 5th and 95th percentiles and outlying points are represented by dots. Black symbols represent maximal aggregation, grey symbols represent final aggregation 5 mins after agonist addition.

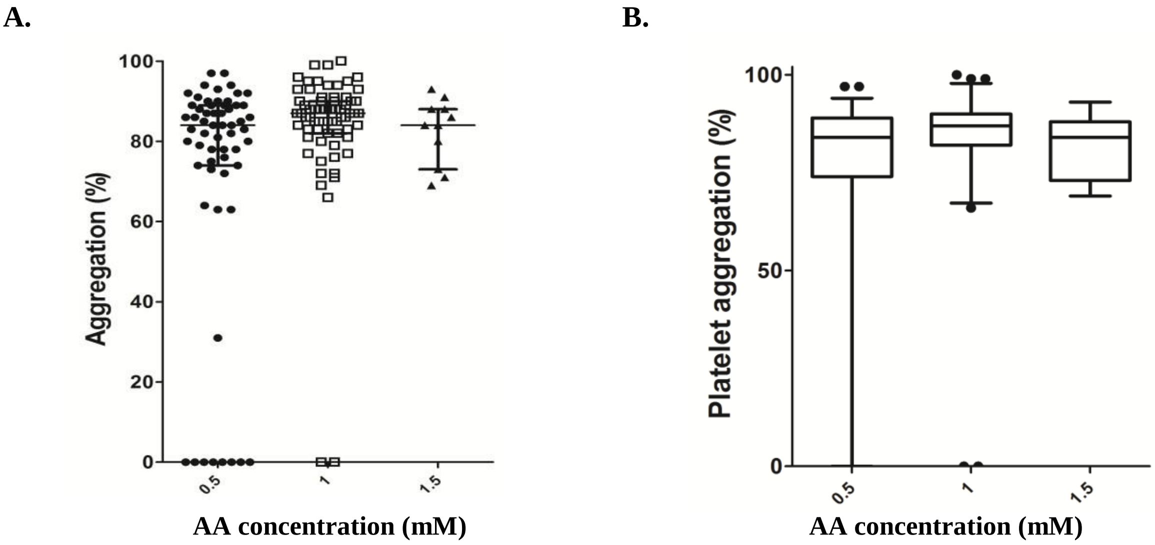

Supplementary Figure 1.3 Arachidonic acid responses. For panel A, bars represent median and interquartile range. For panel B, boxes represent median and interquartile range, whiskers represent 5th and 95th percentiles and outlying points are represented by dots. Maximal aggregation only is shown (as no healthy volunteers showed deaggregation to arachidonic acid).

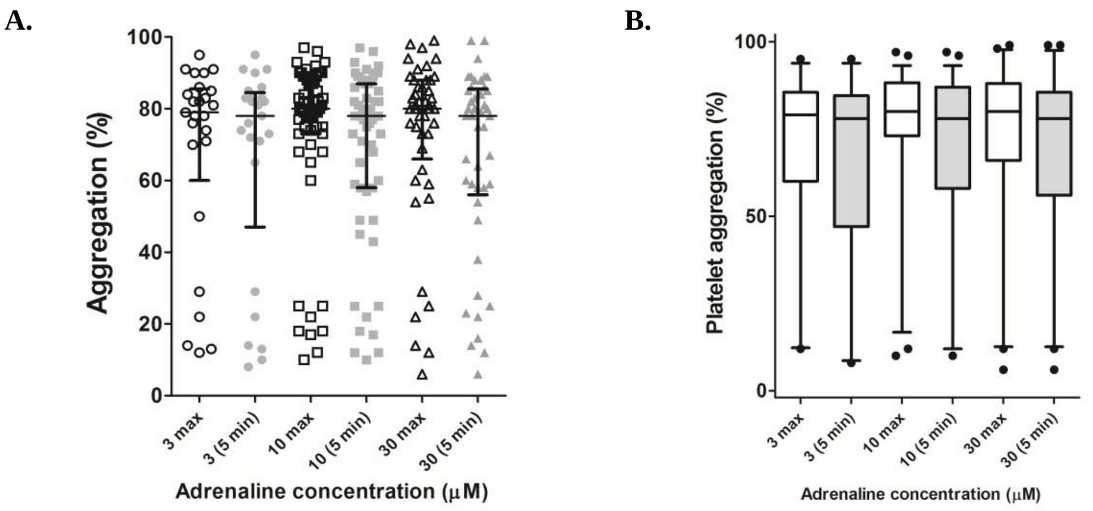

Supplementary Figure 1.4 Adrenaline responses. For panel A, bars represent median and interquartile range. Black symbols represent maximal aggregation and grey symbols represent aggregation 5 minutes after agonist addition (aggregation was monitored for ten minutes in total for adrenaline). For panel B, boxes represent median and interquartile range, whiskers represent 5th and 95th percentiles and outlying points are represented by dots. Black symbols represent maximal aggregation and grey symbols represent aggregation 5 minutes after agonist addition (aggregation was monitored for ten minutes in total for adrenaline).

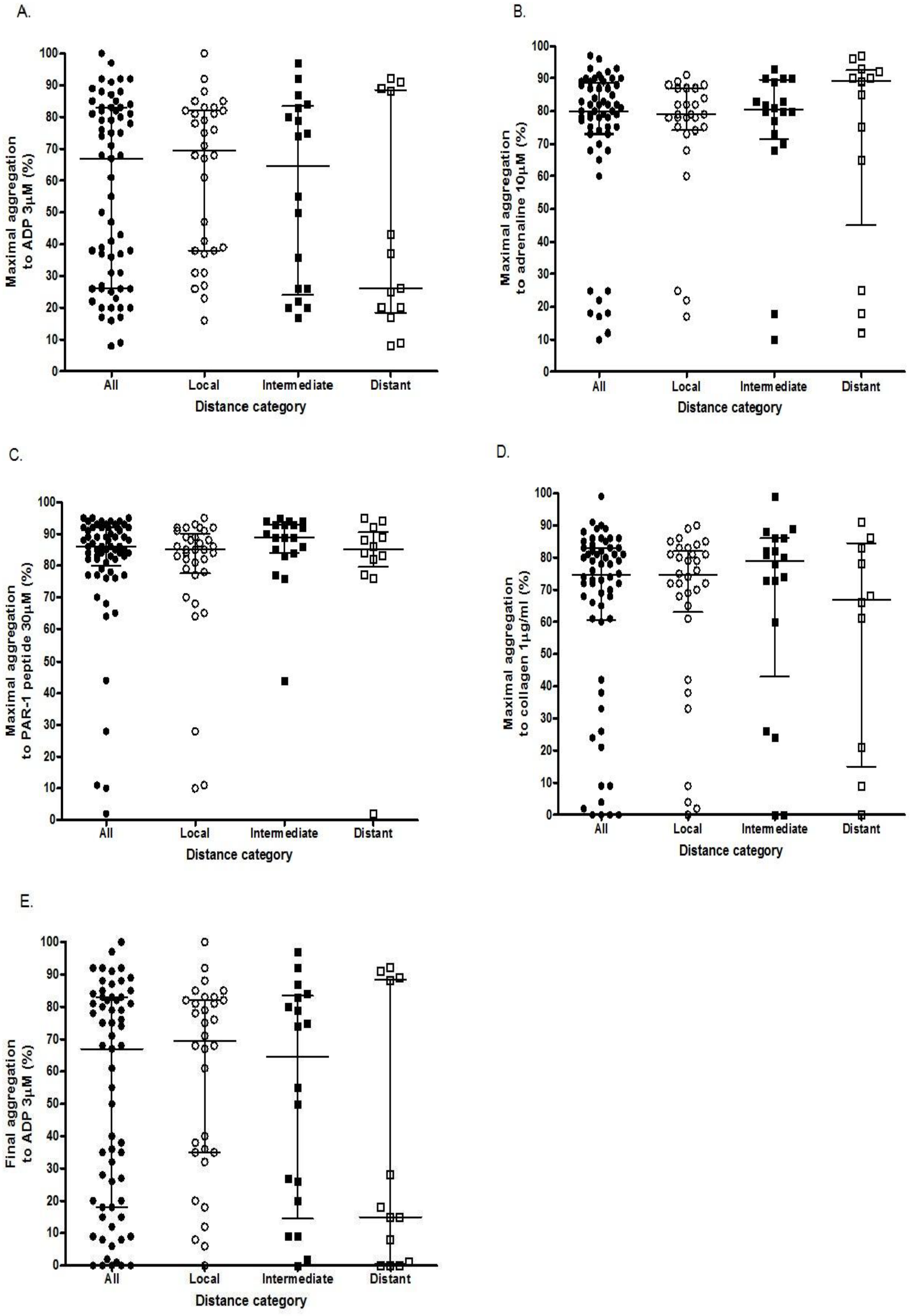
A.
Maximal aggregation to ADP 3μM (%)
Distance category
All
Local
Intermediate
Distant
B.
Maximal aggregation to adrenaline 10μM (%)
Distance category
All
Local
Intermediate
Distant
C.
Maximal aggregation to PAR-1 peptide 30μM (%)
Distance category
All
Local
Intermediate
Distant
D.
Maximal aggregation to collagen 1μg/ml (%)
Distance category
All
Local
Intermediate
Distant
E.
Final aggregation to ADP 3μM (%)
Distance category
All
Local
Intermediate
Distant

Supplementary Figure 2. Effect of distance travelled by sample. X axis for all panels shows all responses, and subcategories of local (testing and venepuncture performed in same city), intermediate (tested from a city less than 100 miles away) and distant (tested from a city 100 miles or more away) testing. Bars represent median and interquartile range. Panel A shows maximal aggregation to ADP 3µM, Panel B shows maximal aggregation to adrenaline 10µM, Panel C shows maximal aggregation to PAR-1 peptide 30µM, Panel D shows maximal aggregation to collagen 1µg/ml, and Panel E shows final aggregation at 5 minutes post agonist addition (allowing for deaggregation) to ADP 3µM. For all panels $p > 0.05$ using Kruskal Wallis test to compare local, intermediate and distant groups. For all panels no p values ≤

Supplemental Acknowledgements

The members of the UK Genotyping and Phenotyping of Platelets Study Group are: Steve P. Watson, Marie Lordkipanidze (University of Birmingham); Andrew D. Mumford and Stuart J. Mundell (University of Bristol); Paul Gissen (University College London); Martina E. Daly (University of Sheffield); Will Lester and Justin Clark (Birmingham Women's Hospital); Mike Williams, Jayashree Motwani, Dianne Marshall, Natalie Lawson, Priscilla Nyatanga, Pat Mann, and Julie Kirwan (Birmingham Children's Hospital); Jonathan Wilde, Tracey Dunkley, Pam Green and April Greenway (University Hospital Birmingham); Michael Makris (Sheffield Haemophilia and Thrombosis Centre, Royal Hallamshire Hospital); Jeanette Payne (Paediatric Haematology Centre, Sheffield Children's Hospital); Sue Pavord, Richard Gooding and Rashesh Dattani (University Hospitals Leicester); Gerry Dolan Charlotte Grimley, Simone Stokley, Emma Astwood, Karyn Longmuir, Cherry Chang, Merri Foros, Michelle Kightley and Linda Trower (Nottingham University Hospitals); Jecko Thachil (previously Paula Bolton Maggs), Charlie Hay, Gill Pike, Andrew Will, John Grainger, Matt Foulkes, and Mona Fareh (Central Manchester National Health Service [NHS] Foundation Trust); Kate Talks, Tina Biss, Patrick Kesteven, John Hanley, Julie Vowles, Lesley Basey, Kevin Knaggs and Michelle Barnes (Newcastle upon Tyne Hospitals NHS Trust); Peter Collins, Rachel Rayment, Raza Alikhan, Ana Guerrero Rebecca Morris, and Dianne Mansell (Cardiff and Vale University Local Health Board); Cheng Hock Toh and Vanessa Martlew (Royal Liverpool University Hospitals); Elaine Murphy and Robin Lachmann (University College London Hospitals NHS Trust); Peter Rose, Oliver Chapman, Anand Lokare, Kathryn Marshall, and Naseem Khan (University Hospitals Coventry and Warwickshire); David Keeling, Nikki Curry and Paul Giangrande (Oxford Radcliffe Hospitals NHS Trust); Steve Austin, David Bevan and Jayanthi Alamelu (Guys'

and St. Thomas' NHS Foundation Trust); David Allsup, Andrew Fletcher, Katherine Gladstone, Jeanette Fenwick, Philippa Woods and Darren Camp (Hull and East Yorkshire Hospitals NHS trust, Castle Hill Hospital, Hull); Beki James, Suzie Preston and Chung Lai-Wah (Leeds Teaching Hospitals NHS trust); Angela Thomas (Royal Hospital for Sick Children Edinburgh); Bethan Myers (Lincoln County Hospital); Gillian Evans, Kim Elliot, Karen Davies, Charlotte Graham and Miranda Foad, (Kent & Canterbury Hospital).

Dr Neil Morgan has authorised authorship on behalf of the UK Genotyping and Phenotyping of Platelets Study Group.
