# Supplementary material for: Investigation of the contribution of an underlying platelet defect in women with unexplained heavy menstrual bleeding
Source: Platelets. 2018 Dec 6;30(1):56–65. doi: 10.1080/09537104.2018.1543865 (PMC6406209; doi:10.1080/09537104.2018.1543865)
Supplement: Supplemental Material [file IPLT_A_1543865_SM0752.zip › supp_mat/Supplemental acknowledgements GAPP study group.docx]

**Supplemental Acknowledgements**

The members of the UK Genotyping and Phenotyping of Platelets Study Group are: Steve P. Watson, Marie Lordkipanidze (University of Birmingham); Andrew D. Mumford and Stuart J. Mundell (University of Bristol); Paul Gissen (University College London); Martina E. Daly (University of Sheffield); Will Lester and Justin Clark (Birmingham Women’s Hospital); Mike Williams, Jayashree Motwani, Dianne Marshall, Natalie Lawson, Priscilla Nyatanga, Pat Mann, and Julie Kirwan (Birmingham Children’s Hospital); Jonathan Wilde, Tracey Dunkley, Pam Green and April Greenway (University Hospital Birmingham); Michael Makris (Sheffield Haemophilia and Thrombosis Centre, Royal Hallamshire Hospital); Jeanette Payne (Paediatric Haematology Centre, Sheffield Children’s Hospital); Sue Pavord, Richard Gooding and Rashesh Dattani (University Hospitals Leicester); Gerry Dolan Charlotte Grimley, Simone Stokley, Emma Astwood, Karyn Longmuir, Cherry Chang, Merri Foros, Michelle Kightley and Linda Trower (Nottingham University Hospitals); Jecko Thachil (previously Paula Bolton Maggs), Charlie Hay, Gill Pike, Andrew Will, John Grainger, Matt Foulkes, and Mona Fareh (Central Manchester National Health Service [NHS] Foundation Trust); Kate Talks, Tina Biss, Patrick Kesteven, John Hanley, Julie Vowles, Lesley Basey, Kevin Knaggs and Michelle Barnes (Newcastle upon Tyne Hospitals NHS Trust); Peter Collins, Rachel Rayment, Raza Alikhan, Ana Guerrero Rebecca Morris, and Dianne Mansell (Cardiff and Vale University Local Health Board); Cheng Hock Toh and Vanessa Martlew (Royal Liverpool University Hospitals); Elaine Murphy and Robin Lachmann (University College London Hospitals NHS Trust); Peter Rose, Oliver Chapman, Anand Lokare, Kathryn Marshall, and Naseem Khan (University Hospitals Coventry and Warwickshire); David Keeling, Nikki Curry and Paul Giangrande (Oxford Radcliffe Hospitals NHS Trust); Steve Austin, David Bevan and Jayanthi Alamelu (Guys’ and St. Thomas’ NHS Foundation Trust); David Allsup, Andrew Fletcher, Katherine Gladstone, Jeanette Fenwick, Philippa Woods and Darren Camp (Hull and East Yorkshire Hospitals NHS trust, Castle Hill Hospital, Hull); Beki James, Suzie Preston and Chung Lai-Wah (Leeds Teaching Hospitals NHS trust); Angela Thomas (Royal Hospital for Sick Children Edinburgh); Bethan Myers (Lincoln County Hospital); Gillian Evans, Kim Elliot, Karen Davies, Charlotte Graham and Miranda Foad, (Kent & Canterbury Hospital).

Dr Neil Morgan has authorised authorship on behalf of the UK Genotyping and Phenotyping of Platelets Study Group.
